# Intensity-specific considerations for exercise for patients with inflammatory bowel disease

**DOI:** 10.1093/gastro/goad004

**Published:** 2023-02-20

**Authors:** Andrew J Ordille, Sangita Phadtare

**Affiliations:** Department of Biomedical Sciences, Cooper Medical School of Rowan University, Camden, NJ, USA; Department of Biomedical Sciences, Cooper Medical School of Rowan University, Camden, NJ, USA

**Keywords:** IBD, strenuous exercise, exercise intensity, Crohn’s disease, ulcerative colitis

## Abstract

The rising prevalence of inflammatory bowel disease (IBD) necessitates that patients be given increased access to cost-effective interventions to manage the disease. Exercise is a non-pharmacologic intervention that advantageously affects clinical aspects of IBD, including disease activity, immune competency, inflammation, quality of life, fatigue, and psychological factors. It is well established that exercise performed at low-to-moderate intensity across different modalities manifests many of these diseased-related benefits while also ensuring patient safety. Much less is known about higher-intensity exercise. The aim of this review is to summarize findings on the relationship between strenuous exercise and IBD-related outcomes. In healthy adults, prolonged strenuous exercise may unfavorably alter a variety of gastrointestinal (GI) parameters including permeability, blood flow, motility, and neuro-endocrine changes. These intensity- and gut-specific changes are hypothesized to worsen IBD-related clinical presentations such as diarrhea, GI bleeding, and colonic inflammation. Despite this, there also exists the evidence that higher-intensity exercise may positively influence microbiome as well as alter the inflammatory and immunomodulatory changes seen with IBD. Our findings recognize that safety for IBD patients doing prolonged strenuous exercise is no more compromised than those doing lower-intensity work. Safety with prolonged, strenuous exercise may be achieved with adjustments including adequate hydration, nutrition, drug avoidance, and careful attention to patient history and symptomatology. Future work is needed to better understand this intensity-dependent relationship so that guidelines can be created for IBD patients wishing to participate in high-intensity exercise or sport.

## Introduction

Inflammatory bowel disease (IBD) is an umbrella term for two separate autoimmune pathologies: ulcerative colitis (UC) and Crohn’s disease (CD), both of which cause chronic inflammation of the gastrointestinal tract. This autoimmune inflammatory response manifests as chronic activation of mucosal immunity and intestinal inflammation [1–3]. The severity of symptomatology associates with extent (focal vs pan) and location (colonic vs anywhere) of inflammation in UC and CD patients, respectively. These differences account for the variability in the clinical course of the disease in IBD patients. Clinical distinctions between CD and UC are also made based on the serological markers (antineutrophilic cytoplasmic vs anti-*Saccharomyces cerevisiae* antibodies), as well as specific architectural alterations to intestinal anatomy (micro- and macroscopic findings) [4, 5]. There also exists an IBD subset termed inflammatory bowel disease undetermined where an adequate differentiation cannot be made based on these findings. Albeit their histopathological differences, CD and UC share several similarities in their clinical presentations, including diarrhea, abdominal pain, weight loss, and extra-intestinal manifestations [6].

The epidemiology of IBD regarding race and ethnicity can be described as a disease of shifting and dynamic distribution. While IBD is still predominantly prevalent within Caucasian and Ashkenazic Jewish ethnicities, recent work has demonstrated that the gap between whites and ethnic minority groups in the USA is decreasing [1, 3, 7]. Despite its dynamic distribution among ethnic and racial groups, IBD is still a disease that affects both men and women equally [2, 3, 8]. IBD is reported to have its highest prevalence rate in North America, with some estimates indicating rates to be as high as 439 cases per 100,000 in the USA [9, 10]. While historically considered a disease of high-income countries, the global prevalence of IBD is increasing as a whole, with a reported 6.8 million cases in 2017 as compared with 3.7 million in 1990 [11]. This global increase can partially be attributed to the increased disease prevalence in previously unaffected low-income geographic areas, which is likely due to a combination of changes in socioeconomic, lifestyle, and behavioral factors [10, 11]. Because of the increasing prevalence of IBD in low-income areas, healthcare leaders need to develop strategies to help mitigate the global healthcare burden of IBD in resource-deprived areas.

Currently, IBD treatment is centered on providing symptomatic relief, inducing long-term remission, and reducing risks incurred by complications of the pathogenesis of the disease. Treatment options include a variety of pharmacological and surgical interventions, which are individualized to varying disease presentations, patient risk factors, and specific patient needs. Mainstays of pharmacological treatment for IBD include antibiotics (ciprofloxacin, metronidazole), anti-inflammatory agents (aminosalicyclates, corticosteroids), immune system suppressors (azathioprine, mercaptopurine, methotrexate), and biologics (infliximab) [12–14]. These pharmacological interventions alter the course of the disease by modifying specific points in the biochemical pathways related to IBD pathogenesis.

Treatment for IBD, along with other IBD-related healthcare costs, contributes to a >3-fold increase in the direct cost of care and twice the amount of out-of-pocket expenses compared with non-IBD patients [15]. The financial burden of managing IBD due to increased disease prevalence will inevitably strain healthcare economic systems. To offset both the direct and indirect consequences of an increased IBD burden in the coming years, strategies need to be implemented. Kaplan *et al*. described recommendations to be implemented over the next 10 years to offset this burden. These recommendations illustrate the need for population-level-based interventions to reduce the risk of IBD onset in addition to minimizing the cost of biologics, reducing environmental exposures, and implementing cost-effective healthcare delivery models [16]. It is also recommended that healthcare providers need to implement additional therapeutic interventions aimed at managing disease onset, providing symptomatic relief, and maintaining quiescent disease status. Some of these interventions should be aimed at increasing access to non-pharmacologic treatment strategies. These modalities include dietary modification, psychological mind-based therapy, and exercise [17, 18]. These modalities offer cost-effective strategies that act at a population level and aid clinicians in offsetting the increasing healthcare burden incurred by IBD. While not a substitute for pharmacologic or surgical interventions, these modalities are supportive in the management of IBD. Physical activity may be viewed as an important non-pharmacological intervention in managing IBD due to its cost-effectiveness and accessibility.

Many psychological, emotional, and physical benefits exhibited in IBD patients are with low-to-moderate exercise interventions. While numerous studies have corroborated the advantageous effects of lower-intensity exercise on IBD-related health parameters, there is significantly less focus on the effects of high-intensity exercise on IBD outcomes. This gap in knowledge is significant and needs to be filled, as a fraction of the IBD patient population may actively engage in strenuous exercise without any definitive understanding of the risks and benefits. To the best of our knowledge, no available summary or consensus on intensity-specific considerations exist for IBD patients wishing to exercise effectively and safely. This review is the first attempt to summarize findings on the relationship between strenuous exercise and health-related outcomes in IBD patients. In this article, we discuss the current exercise guidelines for the IBD patient and the correlation between low-to-moderate vs strenuous exercise regimens and IBD symptom manifestation. Moreover, we discuss molecular mechanisms underlying the modulatory effects of exercise on the dysregulated inflammation seen in IBD patients. We also comment on the patient preferences towards exercise modalities.

## Search Strategy

###  

We conducted a wide-searching literature review to identify articles that addressed the mechanisms and outcomes of various exercise intensities in IBD patients. The following search engines and electronic databases were accessed for articles published up until August 2022: MEDLINE (access via PubMed), Embase, and Google Scholar. Using Boolean operators and Medical Subject Heading (MeSH) terms, the following search terms were entered into each database with various phrase inputs: inflammatory bowel disease, CD, ulcerative colitis, exercise, exercise intensity, high intensity, strenuous exercise, vigorous intensity, physical activity, exercise, health outcome, and disease activity. All the papers obtained by our search strategy were individually reviewed for their content and relevance to the aims of this review. We also read additional papers that came up as the most relevant in the respective references of above articles.

The inclusion criteria of the studies were as follows: (i) randomized–controlled trials and cross-sectional studies that addressed the study aims; (ii) original studies and review articles that provided context or understanding of exercise-related changes in IBD patients; (iii) accessible in English language; and (iv) focused on either pediatric or adult patient populations. Exclusion criteria were limited to (i) studies not in English language; (ii) articles that could not clearly identify IBD disease activity or outcomes; and (iii) studies that did not attempt to delineate exercise intensity as a variable.

## Exercise recommendations and physical activity associations

### The correlations between exercise, activity levels, and the prevalence of IBD

The potential protective effects of physical activity in IBD patients have been described in the literature. Cucino *et al*. [19] used data from the National Center for Health statistics to show that significant correlations between IBD mortality and occupation existed. Specifically, IBD mortality was significantly reduced for patients with occupations of manual labor, including farmers and laborers, and increased in those with sedentary jobs. Early IBD demographic studies in Germany and Denmark build upon this relationship, with data revealing associations between labor-intensive occupation and reduced disease prevalence [20, 21]. These studies indicate the protective effects of exercise on disease onset. Contrary to these findings, Narula and Fedorak stated that there is no support for an occupation–disease onset relationship in the IBD population due to inconsistencies in results reported [22]. Regardless, these early studies sparked an early debate that physical activity levels may contribute to IBD pathogenesis in some way.

A study by Klein *et al*. [23] examining pre-illness factors and their contribution to IBD disease onset observed that patients had lower levels of activity during this period as compared with controls (*P *<* *0.001). A postal survey of Stockholm county residents with CD by Persson *et al*. [24] revealed that daily exercise was protective for IBD patients [relative risk: 0.6; 95% confidence interval (CI): 0.4–0.9]. A case–control study by Oravcova *et al*. [25] of 338 Slovakian IBD patients compared with matched controls showed there to be an association between UC and minimal weekly sporting activities in childhood [odds ratio (OR): 2.0, 95% CI: 1.1–3.5, *P *=* *0.02]. A prospective study of 194,711 women from a Nurses’ Health Study examined the effects of physical activity on CD and UC risks. Findings demonstrated an inverse risk of disease with physical activity. Furthermore, the results revealed that women obtaining at least 27 metabolic equivalents task (MET) hours per week had a 44% reduction in risk for developing CD compared with sedentary women (<3 MET hr/week) [26]. A prospective study of patients based on self-reporting showed that CD patients with higher exercise levels were less likely to have active disease at the 6-month point of study surveillance [27]. Given the abundance of data supporting physical activity’s protection against IBD, research efforts have focused on elucidating the effects that exercise has on IBD pathogenesis.

### Exercise recommendations for healthy adults

Exercise has long been appreciated as a mechanism by which we improve our longevity, functional capacity, and psychological well-being [28]. Some of these adaptations are incurred through biological changes, including improved lipoprotein profiles, coronary blood flow, endothelial function, and adiposity storage [29–32]. Exercise has also been used as a tool for evading disease, which is evidenced by improvements in cardiovascular- and cancer-related mortalities in physically active individuals [33]. As an attestation to its importance, the US Department of Health and Human Services recommends that persons between the ages of 18 and 65 years old obtain 150–300 minutes per week of moderate-intensity aerobic exercise, or 75–150 minutes per week of vigorous-intensity aerobic exercise. Additionally, muscle-strengthening exercises should be incorporated for at least 2 days per week [28]. There also exist subset-guidelines for other healthy populations, including children and pregnant women [28]. In addition to having extensively documented effects in healthy individuals, exercise has therapeutic effects in patients managing chronic diseases, including cancer, metabolic disorders, hypertension, cardiovascular disease, chronic obstructive pulmonary disease, and depression [34–39].

### Exercise recommendations for IBD patients

**Figure 1.**
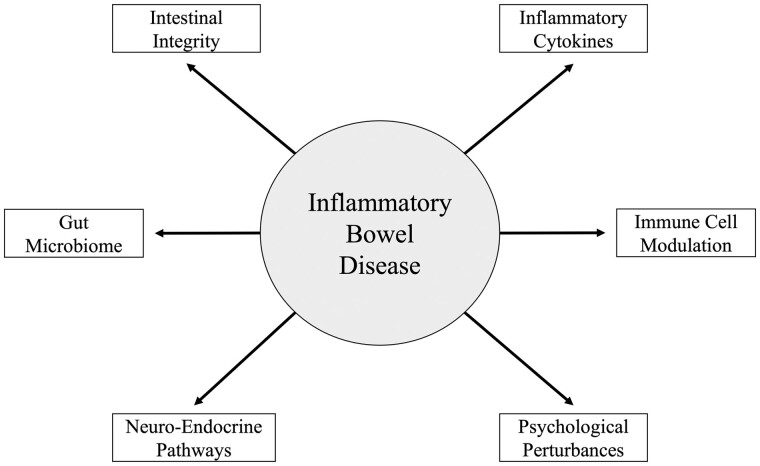
Clinical end points of IBD that are hypothesized to be modified by increased physical activity or exercise.

IBD is a chronic disease that can also be non-pharmacologically managed with exercise (Figure 1). Prior to 1998, many of the exercise recommendations for the IBD population were extrapolated from general population guidelines. A report from Ball in 1998 was the first attempt at providing a comprehensive exercise framework for IBD patients. It put forth precautions, potential complications, and exercise guidelines for the IBD population, stating that programming should include 20–60 minutes of aerobic activity at 50%–85% of maximum oxygen uptake (VO_2_ max) 3–5 days per week in conjunction with resistance training at a minimum of twice per week [40]. Safety-related comments were provided, stating not to exercise during an acute flare-up and waiting at least 2 days until symptoms have subsided to safely participate again. Additionally, it was noted to stay within a person’s targeted training range and to not be an “intensity violator” [40]. This statement is the first to heed caution to the potential deleterious effects of more strenuous exercise intensities in IBD patients, although no underlying biological explanation was provided. The British Society of Gastroenterology stated that while it is difficult to make clear recommendations specifically related to bone health for IBD patients, exercise should be utilized as a tool for the prevention and treatment of osteoporosis in IBD [41]. National organizations currently supporting patients with IBD, including the Crohn’s Colitis Foundation and Crohn’s and Colitis Canada, provide general statements advocating for patients to engage in low-to-moderate exercise modalities, including walking, treadmill running, bicycling, and swimming. Despite the advocacy among these organizations, most studies report that individuals with IBD are not meeting their recommended physical activity guidelines [42]. An online survey of UK adults with IBD showed that only 17% of people reported their activity has “high,” whereas 50% and 33% described their activity levels as “minimally active” or “inactive,” respectively [43]. Objective interpretations of physical activity levels in the IBD population have also been made using accelerometry data, with findings showing that CD patients were more sedentary (97.7% vs 96.2%) and engaged in significantly less moderate-to-vigorous-intensity exercise bouts (1.0 vs 5.0) over a 7-day period compared with healthy matched controls [44].

While other chronic disease populations receive specific and tailored guidance regarding exercise prescription, IBD patients receive guidance that is congruent with recommendations for the general population. The consensus for individuals with IBD wishing to participate in exercise for health-related outcome improvements is to safely participate in low-to-moderate-intensity aerobic exercise in conjunction with other exercise modalities, including strength training, stretching, and yoga. Either due to caution or lack of published literature, guidance available for IBD patients wishing to participate in higher-intensity exercise modalities, with respect to safety, benefits, and harms, is minimal.

## The molecular mechanisms, perturbances, and inflammation in IBD with respect to exercise

### Dysregulated inflammation and physiologic perturbations to the intestines are hallmarks of IBD

The mucosal damage and dysfunctional activation of immune system modulators in the pathogenesis of IBD are mediated by a variety of pro-inflammatory molecules and pathways, contributing to alterations in both innate and adaptive immunity. Fundamental innate immune system changes are represented by polymorphisms in toll-like-receptor function and expression, with subsequent alterations in intestinal microbiota make-up [45, 46]. Cellular degradation processes, including autophagy, are also evidenced to be dysregulated, contributing to an imbalance in cellular homeostasis [47, 48]. Also heavily evidenced is the unfavorable activation of nuclear factor kappa B within intestinal epithelial cells, which also leads to alterations in mucosal regulatory mechanisms and activation of pro-inflammatory cytokine cascades [48, 49].

While these innate immune system changes are regarded as important, much focus has been on the adaptive immune system, as it appears to be the main driver of IBD-related damage and dysregulation. Historically, the CD4^+^ T-helper (Th-) lymphocyte predominance of CD and UC have been attributed to Th-1 and Th-2 effector subsets, respectively. The enhanced Th-1 phenotype observed in CD results in increased levels of cytokines interleukin (IL)-2 and interferon gamma (INF-γ) [50]. Consequently, increased INF-γ from this Th-1 response stimulates macrophages to produce the pro-inflammatory cytokine tumor necrosis factor alpha (TNF-α) [51]. With UC, a Th-2 phenotype is manifested by increased levels of IL-13 [52]. Recent studies have begun to unravel the overlap in Th-cell predominance that exists between these two etiologies [49]. Despite the immunological differences and CD4^+^ subset predominance, both CD and UC result in dysregulated elevations of inflammatory cytokines, including TNF-α, as compared with healthy individuals [51]. New and emerging research has shed light onto the significance of Th-17, the highly pro-inflammatory and autoimmune-inducing CD4^+^ subset that is observed in both CD and UC [53]. Recruitment of this CD4^+^ subset is induced by IL-6, transforming growth factor beta, and IL-23, a cytokine that is critical for mounting early responses against pathogens [53, 54]. IL-23 has been shown to contribute to intestinal inflammation in murine animal models and polymorphisms have been observed in UC and CD populations [48, 55]. Downstream effects of enhanced Th-17 expression include transcription of IL-17, a pro-inflammatory regulator of intestinal inflammation in both CD and UC patients [56].

### What precipitates the disease dysregulation?

The immunologic changes at the core of dysregulation in IBD are more protrusive during times of increased disease activity and are due to internal or external perturbances. Environmental and physiologic factors have been described as triggers of disease flare-ups, including antibiotics, non-steroid anti-inflammatory drugs, diet, and pollutants [57]. Additionally, psychological factors appear to contribute. A multivariate logistic regression from population-level data by Bernstein *et al*. [58] showed that high levels of perceived stress were significantly associated with increased risk of disease flare-ups (OR: 2.40, 95% CI: 1.35–4.26). Exercise is a modality that has been extensively shown to reduce psychological strain in part through the attenuation of stress-hormone pathways. This positive alteration to a clinical end point of IBD serves as an example of the potential ability of exercise to modulate disease activity.

### Exercise modulates immune responses in the general population

**Figure 2.**
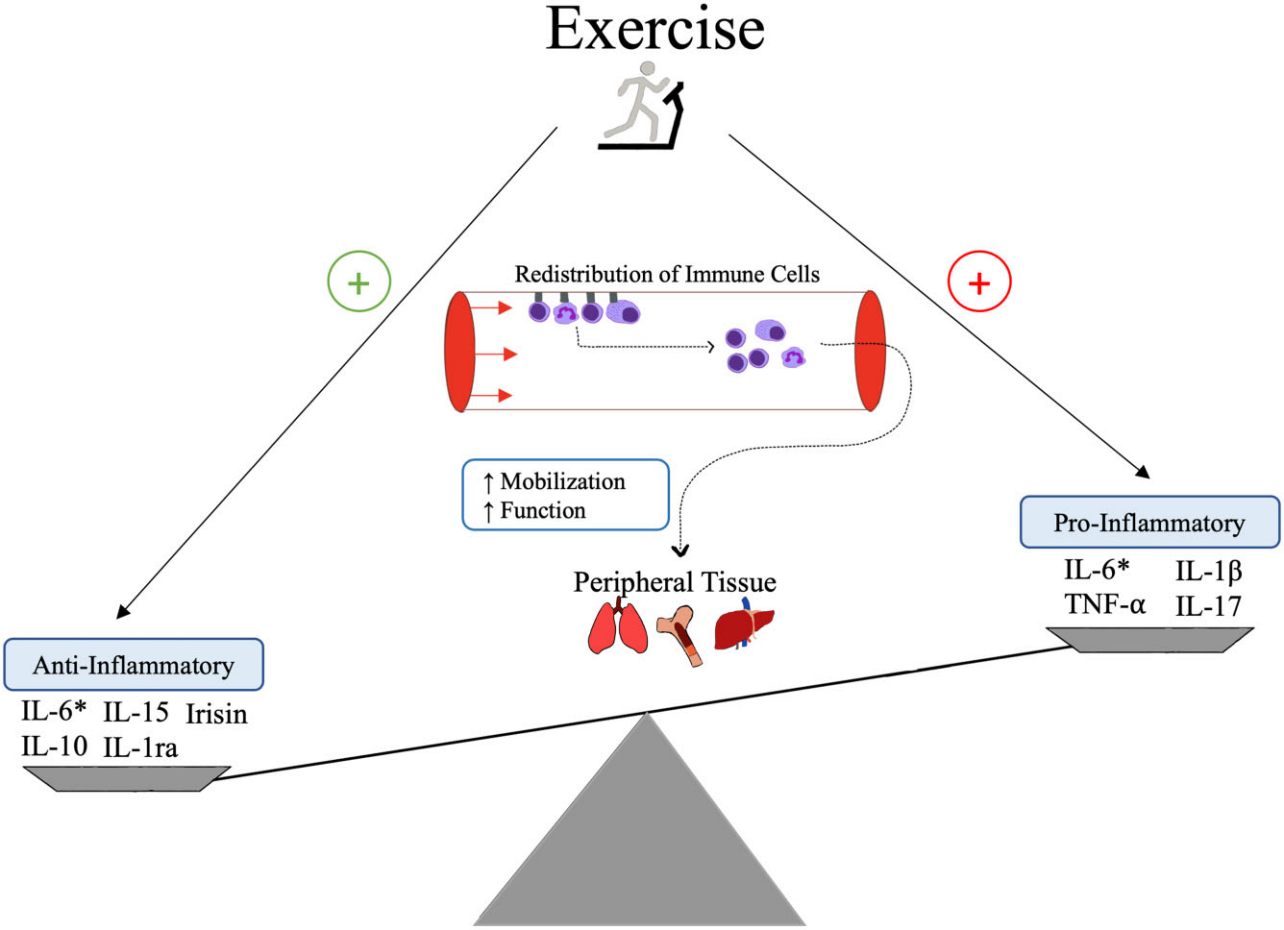
Representation of the inflammatory cytokine and immune cell changes with exercise. The redistribution of immune cells from circulation into peripheral tissues follows an explanation by Campbell *et al.* [70]. *, Interleukin (IL)-6 has dual function as a pro- and anti-inflammatory cytokine. When serving as an anti-inflammatory myokine, IL-6 shifts the inflammatory balance towards an anti-inflammatory state; TNF, tumor necrosis factor.

Molecular changes have been studied to quantify the relationships between physical activity and immunological adaptations seen in exercising individuals. Exercise has been shown to alter the balance of pro-inflammatory/anti-inflammatory states, cytokine release, and crosstalk between biological systems in IBD patients [59]. Generally, exercise is reported to be inversely correlated with systemic inflammation, suggesting an anti-inflammatory effect [60] (Figure 2). Extensive research has documented the favorable immunologic effects of physical activity, including decreased release of pro-inflammatory cytokines, increased levels of circulating IL-6, and subsequent release of trophic factors involved in the repair of intestinal mucosa [61]. Many of these immunological changes are intensity- and duration-dependent. The impact of exercise on immunologic function is further evidenced by animal models, which also show decreased pro-inflammatory cytokines, lymphocytosis, and antibody expansion shortly after exercise [62, 63].

While exercise promotes a general mobilization of leukocytes, there are reports of distinct and immunological profile changes that are intensity-, duration-, and time-dependent. Short-duration (<60 minutes) exercise regimens have demonstrated increased macrophage activity and preferential movement of natural killer and CD8^+^ T lymphocytes during and after exercise [59, 64–66]. Acute exercise regimens of longer duration and higher intensity result in a suppression of innate and adaptive immune system function cell proliferation, immunologic cell function (natural killer cells, T lymphocytes, neutrophils), and elevation of pro-inflammatory cytokines [67–70]. These intensity-specific observations suggest a counterbalance of effects seen with exercise and immune function. While improvements in overall immune competency are evidenced by frequent habitual training, it also is reported that there is a period of increased infection risk and transient immune suppression immediately following an acute strenuous exercise event [59]. However, recently it has been suggested that this immunosuppressive effect is not likely to be seen with any form of acute exercise, including vigorous-intensity exercise. Rather, evidence of intensity-dependent redeployment of highly functioning lymphocytes into peripheral tissue, where immune surveillance is conducted, favors the notion of a state of enhanced immune competency and regulation [70]. The transient lymphopenia and subsequent immunosuppression that are incorrectly reported in the hours following acute strenuous activity does not appropriately account for the downstream effects of increased exercise intensity, including adrenergic-dependent endothelial detachment of lymphocytes and subsequent mobilization of effector lymphocytes due to intensity-dependent circulatory sheering forces within the circulation [70].

### What are the relevant immune responses modulated by exercise in the IBD population?

While many of the exercise-related IBD improvements remain unclear, most explanations are centered on the therapeutic anti-inflammatory effects of exercise in a pro-inflammatory disease population. Many of these anti-inflammatory effects have been reported to come from myokines, which are exercise-specific cytokines that are released from myocytes during muscle contractions. In addition to exerting anti-inflammatory effects locally, myokines can influence other target tissues via autocrine and paracrine mechanisms [71, 72]. Myokines that are upregulated with exercise in healthy individuals have gained attention because they also are implicated in the pathogenesis of inflammation of IBD patients. Most literature focuses on the myokine IL-6, which interestingly has pro-inflammatory effects in the absence of its exercise-stimulated release. Importantly, IL-6 is also implicated in the regulation of IBD-related inflammatory pathways via its trophic effects. The recruitment by IL-6 of trophic factors, like Glucagon-like peptide-1, have protective and regenerative mechanisms in individuals with intestinal disorders [73]. Further corroborating this molecular relation is the evidence of parallel increases in Glucagon-like peptide-1 and IL-6 levels in exercising mice [61]. In addition to IL-6, there are other myokines such as IL-15 and Iris that are suggested to have protective effects on the colon [59].

The heightened attention on the myokine IL-6 is partially attributed to its relationship with a powerful pro-inflammatory cytokine critically implicated in the IBD pathogenesis: TNF-α. In non-exercising or disease states, the responses of IL-6 appear to be directly associated with plasma TNF-α levels, as seen with sepsis [74]. However, TNF-α levels do not appear to increase in parallel with the exercise-induced increase in myocyte-derived IL-6, indicating that IL-6 can exert superseding anti-inflammatory properties [72, 74]. This finding provides evidence of the biphasic nature of IL-6 as either a pro- or anti-inflammatory cytokine, which in part is driven by exercise or its lack thereof. How the relationship between IL-6, TNF-α, and exercise is specifically applied to IBD patients is still unknown. Future investigations should focus on finding a therapeutic range of IL-6 for IBD patients and mechanisms for sustaining that level. This will allow patients to attenuate the pro-inflammatory effects of TNF-α and evade subsequent disease pathogenesis.

Additional molecular evidence endorsing the colon-protective effects of exercise in IBD patients is seen in animal models. Goetz *et al*. reported that after participating in a 16-week wheel running program, mice had higher IL-10 and lower TNF-α levels, representing an anti-inflammatory effect [75]. A similar experiment whereby obese rats were subjected to high-intensity interval training showed marked reductions in TNF-α levels in colonic tissue following treatment [76]. The attenuation of TNF-α levels and colitis symptoms was also found in an experiment with wild-type and adiponectin knockout mice following 4 weeks of exercise training [63]. Another experiment subjected rats to 2,4,5-trinitrobenzenesulfonic acid-induced colitis and forced them to run on a treadmill for 5 days per week within a 6-week interval. The colitis-induced rats had substantial improved healing of their colitis that was evidenced by lower colonic expressions of TNF-α, IL-1β, and hypoxia-inducible factor 1-α. Serum levels of TNF-α and leptin decreased and levels of the myokines, IL-6 and irisin increased [77]. Using mice in which colitis was induced by dextran sodium sulfate, Qin *et al.* demonstrated that a 7-week swimming program elicited improvements in similar clinical parameters. Furthermore, these adaptations were greater in mice who swam for greater amounts of time in their swimming sessions (90 vs 60 minutes per day, 5 days per week) [78]. Contrary to the protective effects previously mentioned, experimental animals were not devoid of gastrointestinal symptoms exacerbations. Cook *et al*. showed that forced treadmill running in dextran sodium sulfate colitis-induced mice (40 minutes, five times/week, for 6 weeks) caused increased amounts of diarrhea, pro-inflammatory cytokines, and colitis-induced mortality, indicating a significant exacerbation of colitis-related outcomes [79]. The results of these colitis-induced animal studies implicate the anti-inflammatory effects of myokines while also demonstrating their potential to exacerbate symptomatology.

## Implications of low-to-moderate-intensity exercise for IBD patients

### Exercising with IBD: is moderation the key?

The transition from therapeutic to symptomatic colonic outcomes due to exercise is hypothesized to be intensity-dependent. While many of the exercise-related anti-inflammatory effects are observed in low-to-moderate-intensity work, evidence shows that strenuous/vigorous-intensity exercise may produce an antagonistic effect. A variety of immune function parameters have been studied over the years. Neiman summarized available evidence regarding the immuno-modulating effects of strenuous exercise, including neutrophilia, lymphopenia, altered natural killer cell activity, decreased granulocyte oxidative burst, decreased mitogen-induced lymphocyte proliferation, decreased major histocompatibility complex class II expression, increased pro-inflammatory cytokines, and decreased muco-ciliary clearance [69]. The dichotomy of findings from animal and human studies may indicate that there potentially exists a therapeutic range in which exercise has either protective or deleterious effects, especially in IBD populations.

### Safety considerations for low-to-moderate-intensity exercise in IBD patients

For low-to-moderate exercise to be widely accepted as a therapeutic intervention for IBD patients, its safety needs to be, and has been, unanimously appreciated. Work by Benazzato *et al*. showed that IBD patients had an improvement in intestinal permeability after completing moderate-intensity aerobic exercise as measured by recovery of urinary lactulose compared with control patients (0.033 vs 0.017, *P *=* *0.038) [80]. The absence of negative alterations in disease course with lower-intensity exercise corroborates claims of safety for IBD patients wishing to exercise [80–82]. Interestingly, there is ongoing work to improve safety with exercise in IBD populations via improved physiologic monitoring. Henderson *et al*. [83] showed that volatile organic compounds can be used as a non-invasive measure of inflammation in IBD patients during prolonged moderate-intensity walking, with butanoic acid levels significantly correlating with plasma IL-6. Novel investigations like this may help quantify the intensity and duration of exercise that can be safely completed in real-world settings not previously studied. If methodologies like this prove to be valid, it may spur the genesis of new studies aimed at measuring the anti-inflammatory modulating effects of physical activity in previously impractical settings, such as hiking, ultra-endurance events, and everyday occupations.

### Low-to-moderate-intensity exercise has favorable effects on IBD outcomes

Early attempts to quantify the relationship between low-to-moderate-intensity exercise and IBD were from Loudon *et al.* [84, 85]. This study was followed by a study by Ng *et al*. [84, 85] using similar methodology. These studies enrolled CD patients in a 12-week supervised low-intensity walking program that was completed three times per week. Participants who vigorously exercised were excluded from the study. The walking programs resulted in significant improvements in quality of life, as measured by decreased disease-related dysfunction, alterations in lifestyle, and improvements in cardiorespiratory fitness. Subsequent studies provided further support for the notion that lower-intensity exercise improves quality of various life measures in IBD patients [86, 87]. Exercise at low-to-moderate intensities in IBD patients has shown additional beneficial effects in other health-related domains, including preservation of antioxidant status, maintenance of gut microbiota, reductions in inflammatory markers, as well as improvements in body composition, cardiorespiratory fitness, and bone mineral density [80, 81, 88, 89]. While much of the literature reports findings in adult IBD populations, there is evidence of similar benefits with moderate-intensity exercise in pediatric patients. In a multi-arm 8-week case–control study, Legeret *et al*. [88] showed that prolonged moderate-intensity exercise (60%–80% of heart rate maximum, 30 minutes per day, 5 days per week) in pediatric IBD patients elicited reductions in pro-inflammatory markers. In another arm of this study, Mählmann *et al*. [90] failed to show any improvements in psychological functioning, depressive symptoms, or subjective sleep. However, objective sleep measures significantly improved. Given the accessibility and personal preference of low-to-moderate-intensity exercise among the public, additional studies should continue to investigate and report on the IBD-related improvements observed with lower-intensity exercise, as it will have the greatest potential to influence health-related IBD outcomes.

## Equivocal effects of high-intensity exercise and relevant considerations for the IBD population

### The deleterious effects of prolonged, strenuous exercise on the gastrointestinal system

Strenuous exercise has been shown to cause gastrointestinal changes not frequently seen in relatively lower-intensity exercise, including diarrhea, gastrointestinal bleeding, abdominal pain, and ischemic colitis [91]. The biological mechanisms accounting for these changes include intestinal inflammation and permeability alterations, motility changes, decreased intestinal blood flow, and hormonal alterations [92, 93]. Intestinal inflammation and damage have been measured in exercise trials by the peripheral collection of intestinal fatty acid binding protein. Higher levels of this intestine-derived protein are peripherally collected at higher exercise intensities, indicating that strenuous exercises elicits greater gastrointestinal damage [94]. Exercise-related structural changes to the gastrointestinal system, such as deterioration of the mucosal barrier, contribute to unfavorable intestinal permeability changes. Alterations in intestinal permeability are influenced independently and synergistically by exercise intensity, hydration status, and exercising conditions (i.e. hot, humid environment) [95–97]. These permeability changes allow for the translocation of toxins (i.e. lipopolysaccharides) and foreign microorganisms across the intestinal epithelium. The transit of contents through the bowel is significantly altered with higher exercise intensities. Exercise-induced attenuations in gastrointestinal motility are thought to be related to changes in neural inputs, whereby exercise promotes increased sympathetic output and subsequent inhibition of vagal neuronal pathways [98]. Given that the achievement of higher exercise intensities occurs in parallel with increased sympathetic input, higher-intensity exercise has been shown to decrease gastric motility [99]. Many of the aforementioned cellular changes that occur with increasing exercise intensity are because of splanchnic hypoperfusion. Exercise is a highly energy-dependent activity that redirects the peripheral circulation to active muscle so work can be sustained. As a result, less blood is received by the gastrointestinal system and vulnerable “watershed” areas are susceptible to hypoperfusion and subsequent ischemic colitis [100]. The splanchnic hypoperfusion is proportional to the duration of strenuous activity, as 30 minutes of exercise (at 60%–70% of VO_2_ max) is reported to result in a 30%–60% reduction in splanchnic blood flow whereas longer-duration work can result in reductions of as much as 80% [98]. Despite these data, the frequency of ischemic colitis due to splanchnic hypoperfusion remains unpredictable [101]. The constellation of these gastrointestinal findings is supported by a theory of “exercise-induced gastrointestinal syndrome” proposed by Costa *et al.* [102]. The theory states that as exercise intensity and duration increase, the degree of gastrointestinal impairment increases. These findings support the concerns that the physiologic effects of prolonged, strenuous exercise may exacerbate the pathogenesis of pro-inflammatory gastrointestinal diseases, such as IBD.

### IBD-related functional changes observed with high-intensity exercise

Despite the concerns mentioned above, literature supports the use of strenuous exercise modalities in colonic disease states. For example, previous research has demonstrated the effectiveness of higher-intensity exercise in improving physical function aspects of quality of life (physical functioning, pain, limitations) in patients with colorectal cancer [103, 104]. Given this, it is plausible to suggest that IBD patients may obtain similar benefits with this intervention through interaction with various gastrointestinal and disease-related factors.

One of these factors includes the gut microbiome, which contributes to the functionality and integrity of the gastrointestinal system. The multifaceted effects of the microbiome are further evidenced by its relationship to non-gastrointestinal parameters including mood and cognition [105, 106]. Dysregulation of the microbiome is reported in IBD and interventions exists to positively influence its composition [107]. Information about gastrointestinal microbiome changes that take place in healthy individuals who undergo sustained, intense exercise may benefit IBD patients. Keohane *et al*. [108] showed that intense exercise positively influenced gut microbial parameters, including diversity of species, microbial functionality, and upregulation of metabolic potential. Furthermore, these changes were evident at 3 months of follow-up. Results in low-to-moderate intensities were mixed [81, 109]. Given this, it is possible that enhancements in gut microbial capacity seen may combat some of the harmful effects observed with prolonged, exhaustive exercise [108, 110]. It should be noted, however, that in addition to acute exercise events, gut microbial capacity is also influenced by health status, fitness level, and diet [109, 111].

### Are there immunological changes specific for higher-intensity exercise?

While other disease populations have shown high-intensity exercise to be both safe and feasible, debate exists as to whether diseases with highly pro-inflammatory states, such as IBD, should avoid strenuous exercise [112, 113]. Apprehension for supporting strenuous exercise comes from studies in healthy individuals which show that apoptosis of peripheral blood lymphocytes was present following exhaustive exercise but absent after moderate-intensity exercise, possibly suggesting an intensity-related attenuation of immunologic capacity [114]. Intensity-specific concerns also come from evidence showing that high-intensity exercise (30 minutes at 80% peak VO_2_) induces pro-inflammatory cytokine expression in leukocyte subset cell populations, although anti-inflammatory cytokine expression is also observed [115]. However, a review article by Campbell and Turner provides support to reframe the theory of exercise-induced apoptosis from negative to positive. Specifically, the observed apoptosis is with senescent T cells, which serves as a pruning process allowing for the mobilization of progenitor and new immune cells into the periphery [70]. Ultimately, this exercise-induced process advantageously results in a fitter immune system.

### How are patient perceptions and IBD disease activity affected by high-intensity exercise?

Although sparse, there are randomized–controlled, prospective, and cross-sectional studies that have designated high-intensity exercise as an intervention in the IBD population (Table 1). A multi-armed trial by Bottoms *et al*. [116] and Tew *et al*. [112] subjected CD patients to a 12-week program that included moderate-intensity continuous training (30 minutes at 35% peak power for one event) or high-intensity interval training (1 minute at 90% peak power for 10 events) three times per week.

**Table 1.** Studies evaluating the impact of prolonged, strenuous, or high-intensity exercise on IBD parameters

Study	Study type	Subjects	Intervention(s)	End points	Outcomes	Adverse events
Bottoms *et al.* [116]	Randomized–controlled trial	CD patients with quiescent or mildly active disease (*n *=* *25)	Subjects randomized into moderate- or high-intensity exercise groups (three cycling sessions/week × 12 weeks) MICT: 30 min at 35% peak power HIIT: 1 min sprint at 90% peak power × 10 intervals	Affective valence via FS Enjoyment via PACES Ratings exertion of activity, RPE (RPE-Legs, RPE-Central)	No difference in FS (2.2 vs 2.1, *P *=* *0.25) or PACES scores (99.4 vs 101.3, *P *=* *0.78) between HIIT and MICT RPE-L (5.5 vs 3.3, *P *=* *0.03) and RPE-C (5.1 vs 2.9, *P *=* *0.03) were greater during HIIT than during MICT	–
Tew *et al.* [135]	Randomized–controlled trial	CD patients with quiescent or mildly active disease (*n *=* *36) HIIT: *n *=* *13 MICT: *n *=* *12 Control: *n *=* *1	Subjects randomized into moderate-intensity, high-intensity exercise (three cycling sessions/week × 12 weeks) or control group MICT: 30 min at 35% peak power HIIT: 1 min sprint at 90% peak power × 10 intervals	Cardiorespiratory fitness via O_2_ peak Disease activity via CDAI Fatigue via IBD Fatigue Scale Quality of life via IBDQ	Greater increase in peak O_2_: with HIIT vs with MICT (+2.4 vs +0.7 mL/kg/min) Fecal calprotectin % changes from baseline: HIIT (+12%) vs MICT (+40%) vs control (+25%) CDAI % changes from baseline: HIIT (–20%) vs MICT (+41%) vs control (+35%)	HIIT and MICT groups each had one patient drop out due to disease relapse Four adverse events reported: nausea, vomiting, headache
Hassid *et al.* [117]	Prospective cohort study	Patients with IBD (CD: *n* = 7; UC: *n* = 3)	Voluntary participation in a marathon (*n *=* *1), half-marathon (*n *=* *6), triathlon (*n *=* *1), or bicycle ride of >45 miles (*n *=* *3)	Fecal calprotectin Disease activity for CD via HBI, for UC via Simple Clinical Colitis Activity Index	There were no elevated fecal calprotectin levels before (32 μg/mg), 24 h post-event (23 μg/mg), or 1 week post-event No difference in pre- and post-event fecal calprotectin levels	Two CD patients experienced an increase in HBI index (12 to 16, 3 to 7). Both scores returned to at or below baseline levels at 1 week post-event
Taylor *et al.* [104]	Cross-sectional study	Patients with IBD (CD: *n* = 96; UC: *n* = 145; undetermined diagnosis: *n* = 1)	Survey measuring associations of physical activity habits, resilience, and quality-of-life measures by linear regressions	Time spent doing moderate-to-vigorous physical activity (min/week, MET-min/week) via IPAQ HRQoL via the SF-36 Resilience via the 10-item Connor-Davidson resilience scale	Linear regression: moderate-to-vigorous physical activity was associated with physical domains of SF-36 (β = 0.098, *P *<* *0.001) but not mental domains of SF-36 (β = 0.01, *P *=* *0.079) Higher volume of moderate-to-vigorous physical activity (>150 min/week) was significantly associated with improved physical HRQoL, but not mental HRQoL	–
Ploeger *et al.* [122]	Cross-over study	Pediatric patients with CD (*n *=* *15) and healthy controls (*n *=* *15)	Completion of two exercise interventions separated by 1 week MICE: two bouts of 30 min of cycling at 60% peak aerobic mechanical power, W_peak_ (total duration: 60 min) HIIT: six bouts of 4 × 15 sec cycling intervals at 100% W_peak_ (total duration: 6 min)	Responses in immune cells (leukocytes, neutrophils, lymphocytes, monocytes), inflammatory cytokines (TNF-α, IL-6, IL-17), and growth hormones (IGF-1, GH)	MICE in patients elicited greater increases in leukocytes (*P *<* *0.05), neutrophils (*P *<* *0.05), lymphocytes (*P *<* *0.05), monocytes (*P *<* *0.001), and IL-6 (*P *<* *0.05) than HIIE TNF-α was not elevated in patients with MICE or HIIE, and no differences in TNF-α were observed between MICE and HIIE in patients Conclusions from IL-17 responses are limited due to minimal detection	–
DuBois *et al.* [119]	Cross-sectional study	Patients with UC (*n *=* *2,052)	Survey measuring associations of dietary habits, physical activity, and UC-related health outcomes by multi-linear regressions	Physical activity data collected using the Godin-Shephard Leisure Time Activity Index Disease outcomes measured via SCCAI, Short Inflammatory Bowel Disease Questionnaire, and the patient-reported outcomes measurement information system	Multivariable regression: leisure-time activity was inversely related to disease activity (β = –0.108, *P *<* *0.001), anxiety (β = –0.025, *P *=* *0.001), depression (β = –0.025, *P *=* *0.001), fatigue (β = –0.058, *P *<* *0.001), and sleep disturbance (β = –0.019, *P *<* *0.001; positive association was seen with IBD-related quality of life (β = 0.063, *P *<* *0.001) and satisfaction with social role (β = 0.063, *P *<* *0.001) Of these measures (excluding depression), strenuous exercise intensity, compared with moderate and mild intensities, had a greater benefit in health outcomes	–
Fagan *et al.* [42]	Cross-sectional study	Patients with IBD (CD: *n* = 46; UC: *n* = 31)	Survey measuring associations of physical activity levels, quality of life, depression, anxiety, fatigue, and disease activity by multi-linear regressions	Time spent in vigorous-intensity exercise, moderate-intensity exercise, and walking via IPAQ Fatigue measured via MFI and IBD-F Disease activity via HBI, SCCAI, and fecal calprotectin, C-reactive protein, and ferritin levels HRQoL measured via IBDQ	In CD patients, higher levels of vigorous activity were associated with lower fatigue (MFI: *r* = –0.376, *P *=* *0.012; IBD-F: *r* = –0.306, *P *=* *0.043) and higher HRQoL (IBDQ-bowel: *r* = 0.322, *P *=* *0.035; IBD-systemic: *r* = 0.380, *P *=* *0.012) In UC patients, lower physical activity levels were associated with higher CRP levels (*r* = –0.0399, *P *=* *0.029) and HRQoL (*r* = –0.407, *P *=* *0.023)	–
Cohen *et al.* [121]	Descriptive study	NFL players with IBD (CD: *n* = 3; UC: *n* = 2)	Individual commentaries on patient-specific IBD health outcomes	Age of diagnosis, race, football position, symptoms of disease, disease course, and years played in NFL	Five IBD patients were identified over a 9,246 athletes from a 20-year span (2000–2019). Mean number of seasons played was greater (7.3 years; range, 5–11 years) than NFL average (3.3 years) Cohort does not represent all IBD patients in NFL during 20-year span	4/5 players underwent colonic resection during their career
Lamers *et al.* [118]	Cross-sectional study	Patients with IBD (CD: *n* = 176; UC: *n* = 162)	Survey measuring associations of disease activity and physical activity levels by multi-linear regressions	Disease activity via CDAI, SCCAI lame Physical activity levels via Short questionnaire to Assess Health-enhancing Physical Activity	CD had inverse relationship between physical activity levels and disease activity scores (β = –0.406, *P *=* *0.007). No association was found for UC patients (β = –0.006, *P *=* *0.162)	–

CD, Crohn’s disease; CDAI, Crohn’s Disease Activity Index; CRP, C-reactive protein; FS, Feeling Scale; HBI, Harvey Bradshaw Index; GH, growth hormone; HIIE, high-intensity intermittent exercise; HIIT, high-intensity interval training; HRQoL, health-related quality of life; IBD, inflammatory bowel disease; IBDQ, Quality of Life Questionnaire; IBD-F, IBD Fatigue Patient Self-Assessment Scale; IGF, insulin-like growth factor; IL, interleukin; IPAQ, International Physical Activity Questionnaire; MICT, moderate-intensity continuous training; MET, metabolic equivalent of task; MFI, Multidimensional Fatigue Inventory; MICE, moderate-intensity continuous exercise; NFL, National Football League; O_2_ peak, peak O_2_ uptake; PACES, Physical Activity Enjoyment Scale; RPE, rate of perceived exertion; SCCAI, Simple Clinical Colitis Activity Index; SF-36, Short Form-36; TNF, tumor necrosis factor; UC, ulcerative colitis.

As expected, cardiorespiratory fitness improvements were seen with high-intensity exercise but no differences were observed in pleasure and enjoyment with exercise between the two intervention groups (*P *=* *0.25 and *P *=* *0.78, respectively), indicating that high-intensity interval exercise elicited similar responses of satisfaction to moderate-intensity continuous exercise. While statistical analyses were not reported for quality of life or disease activity measures, high-intensity exercise did not show disproportionate changes. A single patient from each treatment group had disease relapse during the intervention [112, 116]. Hassid *et al*. used prospective data in 10 IBD patients undergoing strenuous events (marathons, triathlons, etc.) and assessed disease activities following their event. Although two CD patients experienced increased disease symptoms, their disease activity scales returned to baseline levels 1 week post-event. There were no pre-, intra-, or post-event fecal calprotectin level abnormalities in these patients [117]. These findings, although from a small number of patients, provide evidence that strenuous exercise can be tolerable in IBD patients.

Non-experimental evidence also gives insight into this relationship. A cross-sectional study in IBD patients by Taylor *et al*. [104] examined the relationship between time spent doing moderate-to-vigorous physical activity and quality of life. The study showed that increased time spent participating in strenuous activity (i.e. >150 minutes per week) was significantly associated with improved composite scores of physical functioning (function, limitations, pain, health perceptions) [104]. In a similar cross-sectional study, Fagan *et al*. [42] reported that CD patients (*n *=* *46), but not UC patients (*n *=* *31), with higher levels of vigorous activity had a significant association with lower fatigue and improved health-related quality of life. Of note, the physical activity levels in these IBD patients exceeded the exercise guidelines for the public, which contradicts the typically seen IBD exercise patterns in the literature. Therefore, interpretation of these findings should come with caution, as the measure for physical activity levels may be overestimating exercise prevalence. Using multiple linear regressions and survey data, Lamers *et al*. [118] reported that CD patients with higher physical activity levels had lower reported disease activity. It should only be cautiously assumed that these patients had higher physical activity levels due to more higher-intensity activities. This relationship, however, was not observed with UC patients. Recently, DuBois *et al*. [119] recorded disease-related outcomes and physical activity levels in 2,052 UC patients via survey. The findings showed that strenuous exercise, as compared with moderate or mild intensities, had a greater benefit in UC-related health outcomes, including disease activity index, sleep, anxiety, and fatigue.

In these studies, physical activity was self-reported. Therefore, the accuracy of intensities reported during these activities cannot be assessed. This complicates the generalizability and validity of these survey findings. For example, running is an activity on the Godin Leisure-Time Exercise Questionnaire that is listed in the “strenuous exercise” domain of the survey [120]. As such, it can be challenging to use scales like this as a measurement of varying exercise intensities. For example, running is an activity that can be completed at either strenuous or moderate intensity, and it is often unknown what constitutes vigorous activity to patients completing these surveys. Interpretations of these survey findings should thus be cautiously considered.

### Reporting on IBD patient subsets and exercise conditions

Competitive athletes in sport also represent a subset of IBD patients wishing to participate in more strenuous exercise safely and effectively. Cohen *et al*. [121] offered an annotation on a small subset of National Football League (NFL) players with IBD (*n *=* *5) over the last 20 years who played in the NFL—a demanding sport of high-intensity work. Albeit a small sample, these IBD athletes had playing careers (mean: 7.4 years, range: 5–11 years) that were longer than the average for NFL players (3.3 years). However, most of these players would undergo intestinal surgery during their careers and therefore it is unknown whether their careers contributed to IBD disease progression. It also was unspecified whether participation in sport (in-season vs off-season) correlated with symptoms.

While most studies have focused on IBD outcomes in adult populations, even fewer studies have attempted to understand the effect of higher-intensity exercise in children. Ploeger *et al*. [122] appears to have taken the earliest attempt at this in a cross-over trial whereby 15 pediatric CD patients performed moderate-intensity continuous exercise (30 minutes at 60% peak aerobic mechanical power, W_peak_) and high-intensity interval exercise (15-second intervals at 100% W_peak_ for a total of 6 minutes). While there were no differences in TNF-α observed between the two interventions, moderate-intensity continuous training elicited greater increases in leukocytes, neutrophils, lymphocytes, monocytes, and IL-6 as compared with high-intensity work in pediatric CD patients. However, the exercise-induced patterns of inflammation seen in both groups were equivalent to healthy matched controls, indicating that high-intensity work did not appear to disproportionately increase immune markers. Thus, findings of this study suggest high-intensity intermittent exercise to be safe in the pediatric CD population.

In addition to intensity, the duration of exercise is another important exercise characteristic and should be considered when evaluating the relationship between exercise and IBD outcomes. Although not performed at high intensities, Lamers *et al*. [123] showed that IBD patients sustaining prolonged and repetitive walking events (30–50 km on 4 consecutive days) resulted in similar inflammatory cytokine and fecal calprotectin concentrations to healthy control subjects.

While more work is needed to completely understand this relationship, many of the studies presented here provide evidence that benefits seen with strenuous exercise are at least equivalent to those seen in moderate-intensity exercise, specifically regarding disease activity, enjoyment with exercise, fatigue, sleep, and quality of life (Table 2). However, patients wanting to perform at higher intensity levels should be cautioned, as not much information is available regarding the safety and degree of remission achieved in this disease population. While the physiological changes observed with prolonged, exhaustive exercise certainly have potential to exacerbate IBD symptoms, so far there is no evidence for intensity-related detrimental effects. This may, however, be due to a lack of studies performed to specifically test this effect.

**Table 2.** Summary of intensity-related exercise findings for IBD patients

Intensity^a^	Example of exercise	Effects of exercise (beneficial vs detrimental)	Comments
Low < 3.0 METs	Walking, calisthenics	Improved quality of life Reduced disease activity Reduced inflammatory markers	–
Moderate 3.0–5.9 METs	Light jog, cycling, swimming, low-impact sports	Same as above Improved cardiorespiratory fitness	–
High ≥ 6.0 METs	Prolonged, strenuous events (i.e. marathons, triathlons), high-impact sports	Enhanced microbial parameters Enjoyment with exercise Improved quality of life, physical functioning Reduced fatigue, disease activity Increased intestinal inflammation, permeability and reduced intestinal blood	Prolonged, strenuous exercise should be cautioned for IBD patients with increased disease activity or significant structural alterations to colon anatomy Safe participation may be achieved through behavioral modifications Continued research on supplements, drug development, and intestinal monitoring will improve safety

IBD, inflammatory bowel disease; METs, metabolic equivalent of task; 1 MET = 3.5 mL O_2_ per kg of bodyweight per minute.

a Intensities derived from Garber *et al.* [136].

### A bidirectional relationship exists between exercise and IBD

While much of this article has focused on the effects of exercise on IBD-related health outcomes, it should be stated that this relationship is bidirectional. Gatt *et al*. [124] showed that although the majority of patients considered physical activity to be an important part of their lives (87.5%), the diagnosis of IBD negatively impacted their exercise, as measured by using the Godin Leisure-Time Exercise Questionnaire, with a score difference of 6.94 (*P *=* *0.002) following diagnosis. Reasons for decreased fitness among IBD patients are attributed to bowel incontinence, embarrassment related to symptoms, and physical manifestations of the disease (abdominal pain, joint pain, fatigue) [42, 43]. The reduction in exercise seen in this population may also be due to a diminished physical ability. A multiple linear regression analysis by Lamers *et al*. [118] showed that when CD disease activity was increased, physical activity scores decreased (*P *=* *0.035). Brevinge *et al*. [125] evaluated the cycling capacity of CD patients in remission with varying degrees of colonic resection, with findings revealing a 40% decrease in maximal exercise load in patients with the most colonic resection. These findings corroborate the notion that higher-intensity activity may not be attainable and safe in this population, as overexertion may pose a danger to individuals with a diminished capacity for work.

### Risk mitigation strategies for high-intensity exercise in IBD patients

**Figure 3.**
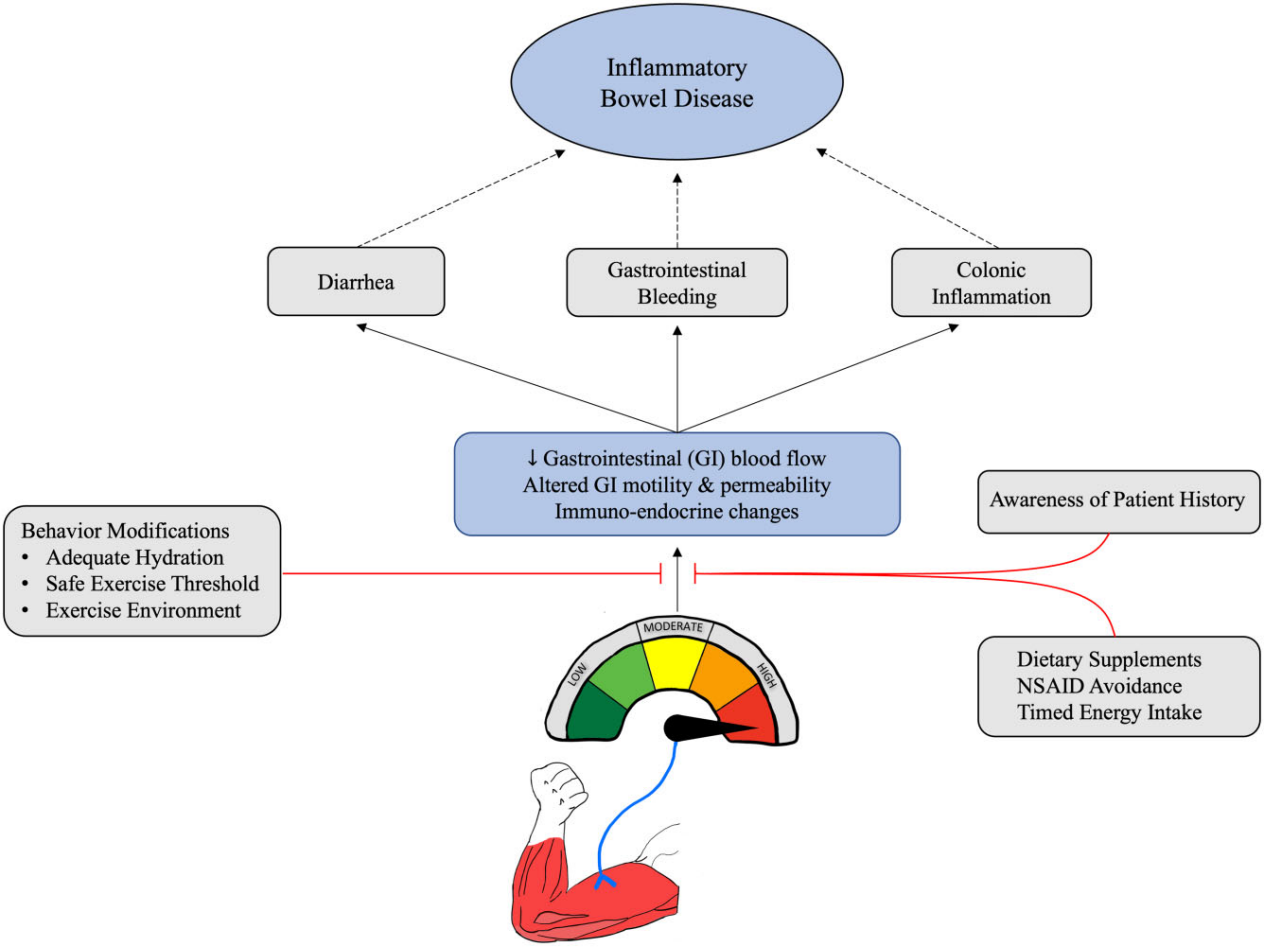
Cascade of changes to gastrointestinal physiology observed with strenuous, high-intensity exercise and the proposed relatedness to inflammatory bowel disease (IBD). Attenuation of these changes may be achieved through specific behavioral modifications, considering a patient’s history, and particular attention to the intake of energy, supplement, and medication. NSAID, non-steroidal anti-inflammatory drug.

For IBD patients wishing to participate in strenuous exercise, many of these risks can be mitigated through behavioral modifications, such as adequate hydration, timed energy intake, and avoidance of non-steroidal anti-inflammatory drugs [92, 126] (Figure 3). Emerging research also supports the use of dietary supplements in augmenting gut function during exercise such as with L-citrulline—an amino acid precursor to L-arginine that is hypothesized to improved splanchnic perfusion and prevent exercise-induced intestinal injury [127]. Although not currently available, a drug that would increase the bioavailability of intestinal nitrous oxide during exercise may provide similar benefits [128]. Furthermore, van Wijck *et al*. [126] suggested a cessation or reduction in exercise intensity when physiological thresholds are reached, but these parameters are not yet defined. A proposed target for such parameters is peripheral monitoring of intestinal fatty acid binding protein—a marker released into the circulation as a result of intestinal damage. A comprehensive patient history might aid in preventing future exercise-related gastrointestinal events. Individuals with a history of gastrointestinal symptomatology during exercise are more prone to reoccurrence of symptoms with increased severity [129]. Therefore, precaution should be provided to these patients, as they are at an increased risk for exacerbations. Finally, disease activity status should be monitored carefully, as patients with active disease status should cautiously avoid additional intestinal stressors during times of heightened symptomatology.

## Application of different training modalities in IBD patients

### The use of resistance training improves IBD-related deficits

A smaller, yet still significant, area of literature regarding IBD-related health outcomes is the use of resistance training. The course of disease in IBD has been reported to affect musculoskeletal function and composition, including strength, lean muscle mass, bone mineral density (BMD), and growth in children [130]. It is postulated that the use of resistance training in IBD populations may ameliorate these changes. Thus far, studies have showed that resistance training is feasible and safe while also achieving observable benefits.

In a letter to the editor, de Souza Tajiri *et al*. [131] reported data which showed that 8 weeks of progressive resistance training in 148 IBD patients with established strength deficits resulted in improved quadricep strength (*P *<* *0.001) and quality-of-life measures (*P *<* *0.001). Other studies have coupled aerobic and resistance training as a therapeutic intervention for IBD patients. Van Erp *et al*. [132] studied 25 IBD patients with severe fatigue who underwent a personalized 12-week exercise program with aerobic and resistance components. These patients showed improvements in maximal power (*P *=* *0.002), fatigue (*P *<* *0.001), and health-related quality of life (*P *<* *0.001). Similarly, Cronin *et al*. [81] enrolled 17 IBD patients into a combined aerobic and resistance training program, with results showing decreased body fat percentage compared with control patients (–2.1% vs 0.1%, *P *=* *0.022). Additionally, this exercise appeared safe, as no deterioration in disease activity was noted (*P *=* *0.435). A randomized–controlled trial of 47 adults with CD by Jones *et al*. [133] further corroborates these findings, showing that a 6-month combined impact and resistance training program (60 minutes per day, three times per week) resulted in improved muscle function (*P *<* *0.001) and greater BMD at the lumbar spine (mean difference: 0.036 g/cm^2^, 95% CI: 0.024–0.048, *P *<* *0.001) as compared with control patients receiving standard care. BMD differences were not observed at the femoral neck (*P *<* *0.059) and greater trochanter (*P *<* *0.415).

The application of this training modality is more congruent with recommendations for healthy adults and is reflective of early frameworks provided for IBD patients [28, 40]. Data thus far suggest that resistance training is both safe and effective for IBD patients. As with all forms of exercise, special attention should be paid to patients with an active disease status, as exercise capacity is limited during this period. Given the benefits observed, reported safety, and alignment with general exercise recommendations, future studies investigating exercise and IBD should include intervention groups that incorporate resistance training so that potential benefits are not underestimated.

### IBD patients exhibit training modality preferences

Physical activity, exercise, and sport are channeled through different activities that are intensity-, geographic-, user-, and culture-dependent. While many of the interventions described in this review utilized aerobic exercise in the form of walking, running, or cycling, activity preferences are not restricted to IBD patients in real-life settings. By use of a survey in a cross-sectional study, Fagan *et al*. [42] reported on the preferred exercise modalities among 77 IBD patients from Dunedin, New Zealand. Walking was the most preferred activity (31%) followed by water-based exercise (18%), high-intensity interval training (13%), cycling (8%), weights (6%), yoga (5%), running (5%), and sports (4%). Preferential participation in walking is supported by other studies [26, 43]. Furthermore, results from a cross-sectional survey by Tew *et al*. [43] report that certain activities, such as running (32% of responses), are preferentially avoided as a result of their IBD.

Interestingly, running has been used as a study intervention for exercise in IBD patients, yet it is not a preferred activity and is even avoided. A possible explanation for this finding is that gastrointestinal symptomatology, such as increased gastrointestinal permeability, in healthy adults is greater with running as compared with other endurance modalities, although mechanisms fully explaining this observation are unknown [92, 95]. Future studies investigating the intensity-specific effects of exercise in IBD patients should acknowledge this observation when designing interventions. Doing so may increase patient participation and satisfaction. Future work should also investigate whether there are IBD-related outcomes due high-intensity exercise that are mediated by specific exercise modalities (running vs cycling vs swimming).

## Conclusions and future directions

Exercise functions as a significant tool in modulating the inflammatory changes and subsequent health-related outcomes in IBD. Reductions in pro-inflammatory cytokines, alterations of the gut microbiota, and improvements in health-related quality of life appear to be outcomes of both low-to-moderate- and high-intensity exercise interventions in IBD patients. While current data support claims of reduced IBD disease activity due to habitual low-to-moderate exercise, sufficient work has not been carried out to describe the effect of high-intensity activity on disease course. As only a few studies show that prolonged, strenuous exercise may also have favorable impacts on IBD outcomes, further investigation is warranted. It is encouraging that adverse events with strenuous exercise modalities have not been disproportionately reported as compared with those with milder activities. While more work is needed to corroborate this notion, strenuous exercise and sport may be safe for IBD patients, especially if protective behavioral modifications related to physical activity are made.

To provide definitive recommendations regarding high-intensity exercise in IBD populations, gaps in knowledge will need to be filled. While limited data from small sample sizes of IBD patients participating in prolonged strenuous exercise events exist, there needs to be a better understanding of how long-term participation in exhaustive exercise impacts IBD disease course. Long-term tracking of physical activity habits with disease activity monitoring can be improved with the use of mobile applications specifically tailored for IBD patients [134]. Analogous to a study by Lamers *et al*., the effects of strenuous exercise should be stratified in IBD patients according to current disease status. Additionally, there are no longitudinal studies that follow disease-remission patients. There are also no studies that compare the intensity of flare-ups by strenuous vs moderate exercise. If it is determined that a positive relationship exists between high-intensity exercise and IBD outcomes, then duration, intensity, and frequency should be determined for maximum benefits. Finally, for IBD patients with careers that require intense involvement in strenuous sport, strategies should be identified for reducing intensity so as not to risk injury or illness. Strategies may focus on real-time monitoring, such as the use of intestinal fatty acid binding protein as a marker of exercise-induced hypoperfusion and cellular damage as suggested by van Wijck *et al*. [126].

## Authors’ Contributions

A.J.O. proposed the idea for the manuscript, carried out the literature search, and drafted the first version of the manuscript. S.P. provided input for writing the manuscript, edited and revised the manuscript, and supervised all parts of the process. Both authors read and approved the final version of the manuscript.
